# Economic evaluation of intensive home treatment in comparison to care as usual alongside a randomised controlled trial

**DOI:** 10.1007/s10198-024-01675-1

**Published:** 2024-04-10

**Authors:** Ansam Barakat, Jurgen E. Cornelis, Jack J. M. Dekker, Nick M. Lommerse, Aartjan T. F. Beekman, Matthijs Blankers

**Affiliations:** 1 Department of Psychiatry Amsterdam UMC/VUmc, Amsterdam Public Health Research Institute Amsterdam UMC, Amsterdam, The Netherlands; 2 Department of Research, Arkin Institute for Mental Health Care, Amsterdam, The Netherlands; 3 Department of Emergency Psychiatry, Arkin Institute for Mental Health Care, Amsterdam, The Netherlands; 4 Department of Clinical, Neuro and Developmental Psychology, Amsterdam Public Health Research Institute, Vrije Universiteit Amsterdam, Amsterdam, The Netherlands; 5 Department of Research and Innovation, GGZ InGeest Specialized Mental Health Care, Amsterdam, The Netherlands; 6 Trimbos Institute, Netherlands Institute of Mental Health and Addiction, Utrecht, The Netherlands; 7 Department of Psychiatry Amsterdam UMC/AMC , Amsterdam Public Health research institute Amsterdam UMC, Amsterdam, The Netherlands

**Keywords:** Economic evaluation, Intensive home treatment, Emergency psychiatry, Pre-randomised controlled trial, H12, I18

## Abstract

**Background:**

There is a dearth of research on the cost-effectiveness of intensive home treatment (IHT), an alternative to psychiatric hospitalisation for patients experiencing psychiatric crises. We therefore present a health economic evaluation alongside a pre-randomised controlled trial of IHT compared to care as usual (CAU).

**Method:**

Patients were pre-randomised to IHT or CAU using a double-consent open-label Zelen design. For the cost-utility analysis, the EuroQol 5-dimensional instrument was used. The cost-effectiveness was assessed using the Brief Psychiatric Rating Scale (BPRS).

**Results:**

Data of 198 patients showed that each additional QALY gained from offering IHT instead of CAU was on average associated with an extra cost of €48,003. There is a 38% likelihood that IHT will lead to more QALYs at lower costs compared to CAU. An improvement of one additional point on the BPRS by offering IHT instead of CAU was associated with an extra cost of €19,203. There is a 38% likelihood that IHT will lead to higher BPRS score improvements at lower costs. Based on the NICE willingness-to-pay threshold of £30,000 (€35,000) per QALY, IHT could potentially be considered cost-effective with a likelihood of 55–60% when viewed from a societal perspective, and > 75% from a health care perspective.

**Conclusions:**

IHT appears slightly more attractive in terms of cost-utility and cost-effectiveness than CAU, although differences in both costs and effects are small especially when viewed from the societal costs perspective. From the health care sector costs perspective, IHT has a higher probability of being cost-effective compared to CAU.

**Trials registration:**

Netherlands Trial Register: NTR6151.

**Supplementary Information:**

The online version contains supplementary material available at 10.1007/s10198-024-01675-1.

## Introduction

Intensive home treatment (IHT) is an alternative to admission to a psychiatric hospital for patients experiencing an acute psychiatric crisis. The development and expansion of IHT has been encouraged in several countries. In the year 2000, IHT was implemented widely in the UK [1], followed by others in Europe such as Norway [2], Switzerland [3], Germany [4] and the Netherlands [5, 6]. Previous studies showed that IHT led to a reduction of acute psychiatric hospitalisation and admission days [1, 3, 7–9] and that it was more acceptable to patients and their families than admission to hospital [6, 8]. Although it is likely that a reduction of hospital admission days leads to cost reductions [7, 10, 11], only one randomised controlled study has assessed the economic impact of IHT, in South London (United Kingdom) [12]. This study concluded that the reduction of inpatient costs was higher than the invested costs for the intervention itself. Although an accumulating number of studies have assessed the effectiveness of IHT in preventing hospital admission and in reducing the number of admission days, there is a lack of randomised trials evaluating the cost-effectiveness of IHT regarding quality of life and psychopathology [7, 8]. We have therefore performed a health economic evaluation of IHT compared to care as usual (CAU) alongside a multicentre randomised controlled trial (RCT).

## Methods

This planned health economic evaluation was performed alongside a two-arm multicentre double-consent open-label Zelen-design RCT to investigate the efficacy of IHT by comparison with CAU. The study protocol was designed in collaboration with clinicians. Input from the clinicians led to the decision to design the RCT as a pre-randomised trial instead of a traditional parallel group RCT. A detailed description of the study protocol, including a health economic analysis plan, has been presented elsewhere [13]. Methods and analyses for the economic evaluation were in line with Drummond et al. [14], reporting guidelines/conventions [15, 16] and previous economic evaluation papers [17, 18]. The study protocol was developed before the latest version of the CHEERS checklist of 2022, so it is unclear what effect methods of engaging patients and others in the study design may have had on the results. The authors assert that all procedures contributing to this work comply with the ethical standards of the relevant national and institutional committees on human experimentation and with the Helsinki Declaration of 1975, as revised in 2008. All procedures involving human patients were approved by the Medical Ethics Committee of VU University Amsterdam (#NL55432.029.16). The trial is registered in the international clinical trials registry platform (NTR6151).

### Participants and procedure

Participants in this study were residents of Amsterdam aged between 18 and 65 years experiencing an acute psychiatric crisis for which hospitalisation was indicated by a psychiatrist. Patients were excluded if they were homeless, had a primary classification of substance-use disorder or intellectual disabilities, lacked basic knowledge of the Dutch language, were currently receiving (Flexible) Assertive Community Treatment care ((F)ACT) [19] or had previously received IHT*.* The enrolment of the patients took place in the two mental health organisations that provide highly intensive psychiatric care through IHT or psychiatric admission in Amsterdam. Patients were recruited by IHT teams and from psychiatric wards between November 2016 and October 2018.

Patients who met the study criteria were pre-randomised to IHT or CAU according to the Zelen double-consent open-label design [20]. In RCTs, patients are ideally allocated in equal numbers to both intervention groups. This is done to obtain the maximum amount of statistical power. In this study, the allocation ratio was planned as 2:1 as a result of staff and facility capacity. Unequal randomisation appears to become more common in clinical trials [21–23]. Before patients were asked for their written informed consent, an assessment of their mental capacity to provide consent for research was conducted by an independent psychiatrist. Patients not considered mentally competent were not included in the study. If patients were willing to participate in the study, they could either participate in the interviews, share their medical records, or both. The analyses presented in this paper are based on the participants interviews, as essential information for the economic evaluation was collected using the interview instruments. Written informed consent was obtained from all patients. Participants were interviewed by trained researchers and were invited for follow-up interviews at six weeks post-baseline interview and at 26 and 52 weeks post-randomisation.

### Interventions

IHT comprises an intensive short-term outpatient mental health care model aiming to minimise hospital admission. IHT is provided by multidisciplinary teams who act as gatekeepers for psychiatric hospital admission by assessing every patient and taking into consideration the necessity of hospitalisation and the possibility of IHT. IHT teams can provide care in two ways: (1) intensive care in the patient’s home setting until the crisis is resolved; or (2) facilitating early hospital discharge by continuing intensive care at home. IHT is provided to vulnerable patients more than twice a week and continues for an average of six weeks until a crisis is resolved.

CAU consists of all commonly available treatments (except IHT). This often includes admission to a mental hospital followed by lower-intensity outpatient mental health care (i.e. less than three times a week). The length of the different phases in CAU depends mostly on the severity of the symptoms, the presence of danger, social factors like housing, and the availability of support from family and friends.

### Resource use and costs valuation

Health care costs included three categories: (1) inpatient mental health care, (2) outpatient mental health care and (3) other care. Inpatient mental health care implies psychiatric hospital admission. Within the participating centres, health care utilisation data were collected from the electronic patient records (EPR). For those participants who reported being hospitalised outside the two participating centres, the non-participating hospitals were asked to share EPR data regarding the frequency and duration of the admission. Outpatient mental health care indicates the types and amount of mental health care received either within the participating centres or elsewhere. Other care included all other forms of health care, including medication. Health care contact data that could not be extracted from EPRs were collected using the first section of the Trimbos Questionnaire for Costs associated with Psychiatric Illness (TiC-P) [24]. This instrument is validated for economic evaluations in populations of psychiatric patients and was administered at baseline and at 26 and 52 weeks after randomisation. The first section of this instrument has a recall period of three months, which was linearly extrapolated to the 6-month period between the consecutive follow-up interviews [25].

Cost prices per dose of medication were extracted from the medication registry [26]. Health care resource utilisation was calculated by multiplying the number of contacts with the reference costs per contact. Costs were valued using standard costs from the Dutch costing guideline [27].

Another source of cost assessed in this economic evaluation was the use of justice system resources. Police and justice-related contacts were recorded during the interviews and valued according to the Dutch justice system costs [28]. In the case of participants who underwent compulsory admission, costs related to judiciary or lawyer’s expenses were evaluated using a report of the Dutch Institute of Mental Health and Addiction [29].

Productivity costs were measured using the second section of the TiC-P, which is the Short-Form Health and Labour Questionnaire (SF-HLQ) [30]. The SF-HLQ assesses whether the participants have been absent from work (absenteeism) or have functioned suboptimally at work due to physical or mental disability (presenteeism). The reported hours/days of lost productivity over the four weeks were extrapolated to the period between the current and previous measurement wave. Productivity losses in hours were multiplied by an estimate of labour costs of €37.90 (men) and €31.60 (women) [30]. Productivity losses were valued using the friction cost method; a maximum friction costs period of 85 days and an elasticity factor of 0.8 were applied.

As the time horizon of the RCT was 52 weeks, no future costs or effects discounting was applied. Dutch unit prices were converted to Organisation for Economic Co-operation and Development (OECD) standard purchasing power parities for the study’s index year, 2019 (104% for the Netherlands), [31] and are expressed in euros (€).

### Effect measures

For the cost-utility analyses, the outcome was the number of quality-adjusted life years (QALYs) gained between the time of randomisation and the follow-up after 52 weeks. QALYs were calculated by weighting the duration of health states with preference-based valuations of health-related quality of life. Using the area under the curve method with linear interpolation, we calculated the number of QALYs gained or lost for each participant. Utilities were determined by measuring the European Quality of Life-5 Dimensions (EQ-5D-5L) at baseline and at 6, 26 and 52 weeks [32]. The Dutch tariff was used to convert the EQ-5D-5L health states to health utility scores [32].

The effect measure for the cost-effectiveness analysis was expressed as a symptomatic outcome using the change in the Brief Psychiatric Rating Scale (BPRS) total score. BPRS scores were collected at baseline and at 6, 26 and 52 weeks. The BPRS is an interview-based instrument consisting of 24 items that can be grouped into four subscales (positive symptoms, negative symptoms, depression and anxiety, disorganisation) and the total sum of all subscales [33]. The total score is the mean of all items and has a range score of 1–7. Higher scores on the BPRS are indicative of greater severity of psychiatric symptoms.

### Data preparations

Multiple imputation (50 imputations) using chained equations [34] was used to replace missing observations in costs and effects data. In the base case analysis, costs and effects data were assumed to be missing at random [35]. We used predictive mean matching to impute missing costs data, as this approach has been shown to work relatively well without transformations even if data are skewed [36], which is often the case for costs data. Rubin’s Rules [37] were applied to pool the outcomes obtained from separate analyses of the 50 multiple imputed datasets. All presented results are based on the multiple imputed data, unless otherwise indicated. We used the R statistical programming environment for all analyses.

### Cost-effectiveness analyses

For all participants who answered the questionnaires, differences in costs and effects between IHT and CAU were calculated as the mean difference in cumulative costs and effects over the 52-week time horizon of this economic evaluation study. Baseline correction was not applied, as randomisation had resulted in sufficient comparability across both interventions at baseline. We extracted a total of 7,500 nonparametric bootstrapped samples from the imputed data. For each of these bootstrapped samples we calculated the incremental costs, incremental effects and an incremental cost-effectiveness ratio (ICER). The ICER indicates the mean additional mental health care costs which have to be spent in the IHT group to gain one additional effect versus the CAU group. Effects were defined as QALYs in the cost-utility analysis and change from baseline in BPRS score in the cost-effectiveness analysis. The ICER was calculated as follows:

$${\text{ICER}} = \frac{{{\text{Costs}}\, {\text{IHT}} - {\text{Costs}}\, {\text{CAU}}}}{{{\text{Effects}}\, {\text{IHT}} - {\text{Effects}}\, {\text{CAU}}}}$$

The ICERs of the 7,500 bootstrapped samples were plotted on cost-effectiveness planes, which present the differences in costs and effects between IHT and CAU in two dimensions by plotting costs against effects. Cost-effectiveness acceptability curves (CEACs) were drawn based on the distribution of the ICERs on the cost-effectiveness planes [38]. CEACs show the probability that IHT is more cost-effective than CAU as a function of the willingness-to-pay (WTP) for one additional QALY or one unit change in BPRS score. Since the WTP is generally unknown, the indifference point is set at a probability of 0.5 on the vertical axis. Above this indifference point, IHT has a better likelihood of being preferred over CAU with regard to cost-effectiveness.

### Base case, alternative scenarios and sensitivity analyses

The base case scenario of this economic evaluation was performed from the societal perspective. In this societal perspective analysis, we accounted for broader costs to society (productivity losses, criminal justice system) in addition to the costs directly related to health care. In an alternative analysis we took the health care sector perspective, in which we accounted only for the costs accrued in the health care sector—as suggested in the CHEERS statement elaboration [15]. There was no statistical power to perform a subgroup analysis due the diversity of the included patient population. To assess the sensitivity of our findings to misspecification of costs for both change in BPRS score and QALYs, one-way sensitivity analyses were performed to evaluate the impact on the ICERs of a − 20 to + 20% misspecification in all cost categories included in the base case analysis.

## Results

### Participants

In total, 246 patients were included in the RCT, 198 (80.5%) participated in the interviews and 48 (19.5%) only gave consent to use their medical records and did not provide data on quality of life and health care, productivity and justice costs. Hence this economic evaluation is based on the 198 participants from whom costs and effects data were obtained. Statistical testing indicated no differences between the interviewed sample (*n* = 198) and the medical records only sample (*n* = 48) regarding age, gender, country of birth, education, marital status, domestic situation, vocational status and the symptom severity and social functioning administered by the Health of the Nation Outcome Scales (HoNOS) at baseline (all ≥ *p* 0.05). The study’s interview compliance rate was high amongst the interview sample, with an overall 87% data completeness rate and a 77% response at 52-week follow-up.

Table 1 presents baseline characteristics of the patients included in the two intervention arms of the study. The majority of the baseline characteristics showed no significant difference between IHT and CAU. In the IHT group, more participants lived with others compared to the CAU group (62.5% vs 42.3%, *χ*^2^ = 6.36, *p* = 0.01).

**Table 1:** Baseline socio-demographics and clinical characteristics of the participating study population

	IHT (*n* = 146)	CAU (*n* = 52)
Age in years; mean (s.d.)	39.72 (12.56)	43.27 (11.99)
Gender; *n* (%)		
Female	83 (56.8)	25 (48.1)
Male	63 (43.2)	27 (51.9)
Country of birth; *n* (%)		
The Netherlands	116 (79.5)	46 (88.5)
Other	30 (20.5)	6 (11.5)
Education; *n* (%)^b^		
Low	13 (9.0%)	6 (11.5%)
Medium	74 (51.0%)	27 (51.9%)
High	58 (40.0%)	19 (36.5%)
Marital status; *n* (%)^b^		
Relationship	52 (35.9)	13 (25.0)
Single, divorced or widowed	93 (64.1)	39 (75.0)
Domestic situation; *n* (%)^c^		
Living with others	90 (62.5)	22 (42.3)
Living alone	54 (37.5)	30 (57.7)
Vocational status; *n* (%)^a^		
Unemployed	70 (48.3%)	28 (53.8%)
Employed	75 (51.7%)	24 (46.2%)
Mental disorders; *n* (%)		
Depressive disorders	33 (22.6%)	12 (23.1%)
Bipolar disorders	31 (21.2%)	12 (23.1%)
Schizophrenia spectrum and other psychotic disorders	52 (35.6%)	16 (30.8%)
Personality disorders	9 (6.2%)	4 (7.7%)
Substance-use disorders	6 (4.1%)	2 (3.8%)
Other disorders	13 (8.9%)	5 (9.6%)
No diagnosis	2 (1.4%)	1 (1.9%)
Duration of care received; mean (s.d.)^a^	84.10 (62.02)	68.02 (77.98)
EQ-5D-5L; mean (s.d.)^d^	0.77 (0.24)	0.79 (0.26)
BPRS total scores; mean (s.d.)^d^	1.81 (0.41)	1.69 (0.35)
Inpatient costs; mean (s.d.)	2850.66 (8769.84)	2917.23 (7047.23)
Outpatient costs; mean (s.d.)	1272.73 (1632.77)	1780.41 (2533.60)
Other health care costs; mean (s.d.)	1951.94 (1906.45)	2367.91 (3059.52)
Productivity costs; mean (s.d.)	5358.24 (7028.68)	5506.00 (7116.42)
Justice system costs; mean (s.d.)	301.79 (866.52)	272.170 (410.58)

IHT indicates intensive home treatment; CAU, care as usual; EQ-5D-5L, European Quality of Life-5 Dimensions, with the Dutch health utilities algorithm applied; BPRS, Brief Psychiatric Rating Scale

^a^Duration of IHT and admission administered in days from baseline to 52 weeks

^b^*n* = 145 (IHT)

^c^*n* = 144 (IHT)

^d^*n* = 48 (CAU) 137 (IHT)

### Clinical outcomes and costs

**Fig. 1:**
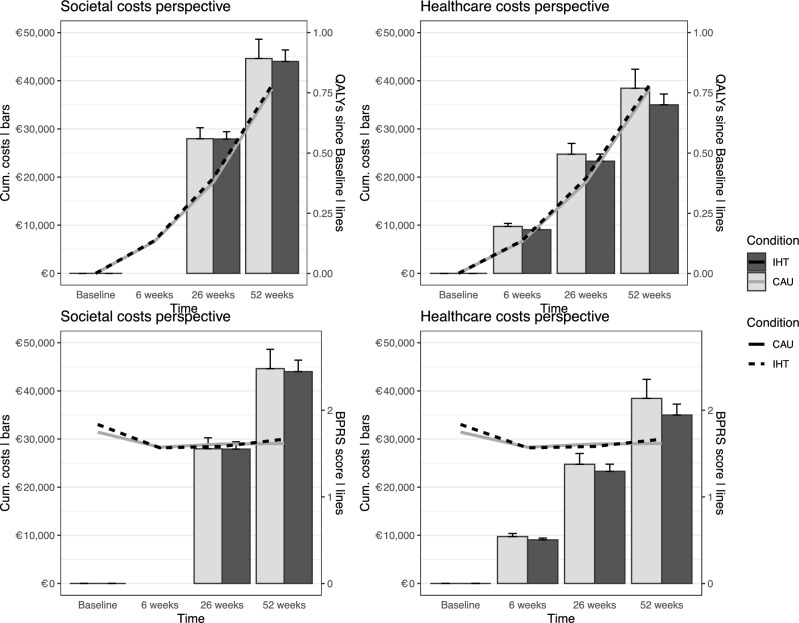
Development of effects and costs from societal and healthcare perspective. Results were based on multiple imputed data. The bars represent the cumulative costs and the lines the effects in quality adjusted life years (QALYS) or brief psychiatric rating scale (BPRS) change from baseline to 52 weeks follow-up. *IHT* intensive home treatment, *CAU* care as usual

The effect parameters of this economic evaluation in the two interventions and from different perspectives are presented in Fig. 1 and Supplementary Material Table 1. QALYs gained during the 52-week follow-up period did not differ significantly between IHT and CAU (mean difference: 0.02; 95% CI − 0.05 to 0.09, *Z* = 0.56, *p* = 0.59). As for the change in BPRS, the BPRS scores in both interventions declined over time, although no significant differences were found between the interventions (mean difference: 0.04; 95% CI − 0.14 to 0.22, *Z* = 0.44, *p* = 0.68).

The difference between IHT and CAU in terms of total societal costs (mean difference of €-557; 95% CI − 9923 to 8237, *Z* = 0.12, *p* = 0.92) and total health care costs (mean difference of €-3,383; 95% CI − 12,542 to 5252, *Z* = 0.74, *p* = 0.47) during the 52-week follow-up period was not significant.

### Cost-utility analysis

**Fig. 2:**
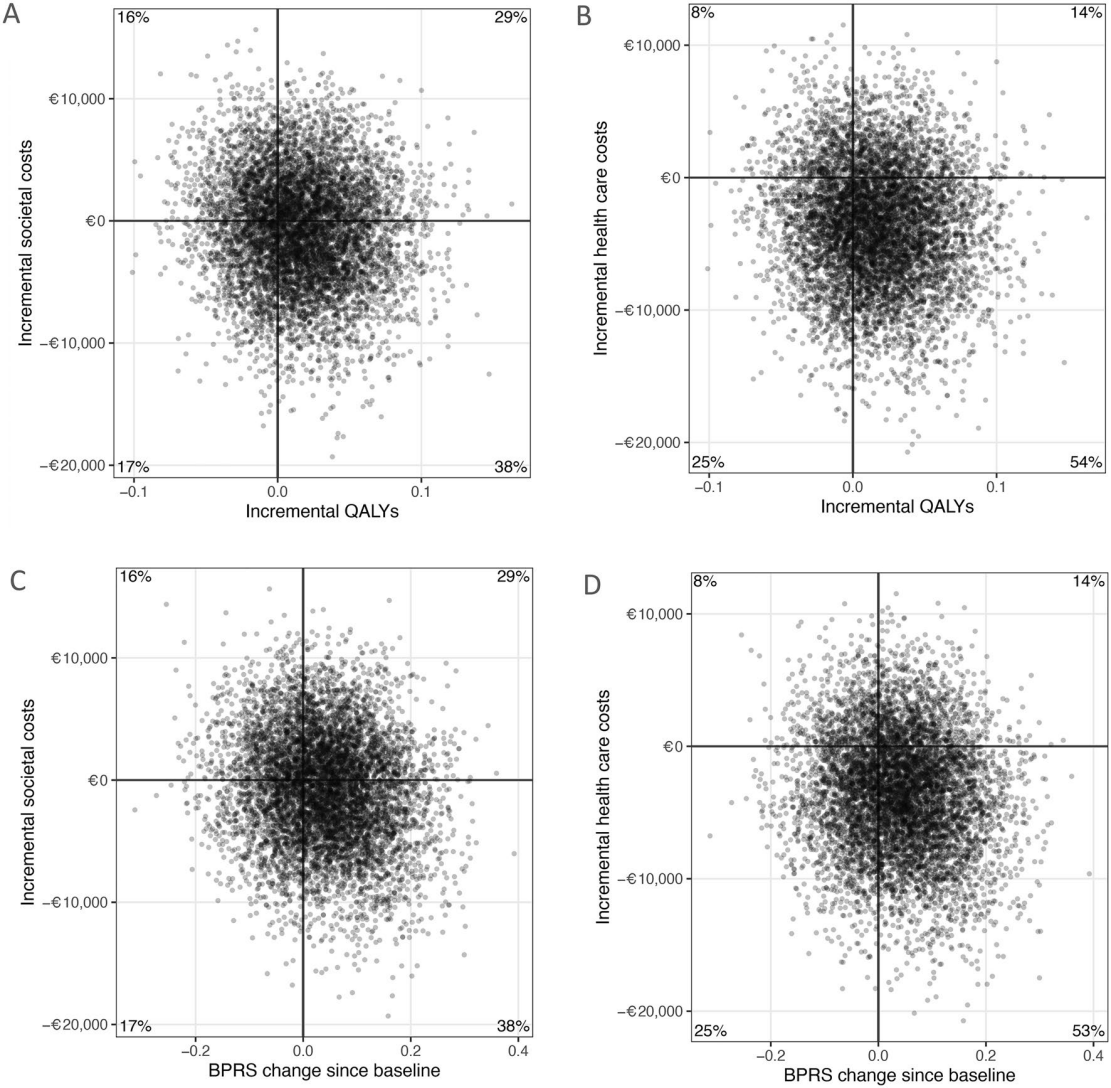
Cost-effectiveness planes. Cost-effectiveness planes showing the change in quality adjusted life year (QALY) or brief psychiatric rating scale (BPRS) during 52 weeks follow-up of the IHT versus the CAU group from a societal and healthcare perspective. In these planes, the horizontal axis indicates differences in health gains between IHT and CAU, while the vertical axis represents differences in costs. The dots indicate the bootstrapped cost-effects pairs which together reflect the uncertainty surrounding the incremental cost-effectiveness ratio (ICER). The chart area is divided into quadrants, each with a specific interpretation

The cost-utility analysis from a societal perspective resulted in a bootstrapped incremental cost-effectiveness ratio (ICER) of €48,003. This indicates that gaining one additional QALY with IHT vs CAU is associated with a median total cost of €48,003. Figure 2A and B show the cost-effectiveness planes under the base case scenario of the cost-utility analysis, by plotting the costs against effects on the graph from the two perspectives. From the societal perspective (Fig. 2A), there was a 38% likelihood of IHT dominating CAU, which means that it would lead to more QALYs at a lower societal cost per participant.

The cost-utility analysis from a health care perspective resulted in a bootstrapped ICER of €-22,759. Disregarding all other costs and focusing only on health care costs, this means that gaining one additional QALY is associated with a median cost saving of €22,759 in the IHT group in comparison with the CAU group. As presented in Fig. 2B, there was a 54% likelihood of IHT dominating CAU, indicating a 54% probability that the intervention would lead to additional QALYs at a lower cost compared with CAU.

### Cost-effectiveness analysis

From a societal perspective, the cost-effectiveness analysis resulted in a bootstrapped ICER of €19,203, which indicated that one extra point improvement in BPRS score was achieved using IHT at an additional societal median cost of €19,203. The cost-effectiveness planes represent the differences in costs and change in BPRS as shown in Fig. 2C. On the basis of the four quadrants, it can be observed that the pattern of the results is similar to those of the cost-utility analyses. A likelihood of 38% is observed in the south-east quadrant, where IHT dominates CAU as it leads to a stronger improvement in the BPRS at lower societal costs.

From the health care perspective, the cost-effectiveness analysis resulted in a bootstrapped ICER of €-8,028. The pattern of results of the cost-effectiveness planes (Fig. 2D) was again comparable to the cost-utility analysis from a health care perspective. There was a 53% likelihood of IHT dominating CAU, which means that it would lead to more improvement in the BPRS at lower costs per participant.

### Cost-effectiveness acceptability curves

**Fig. 3:**
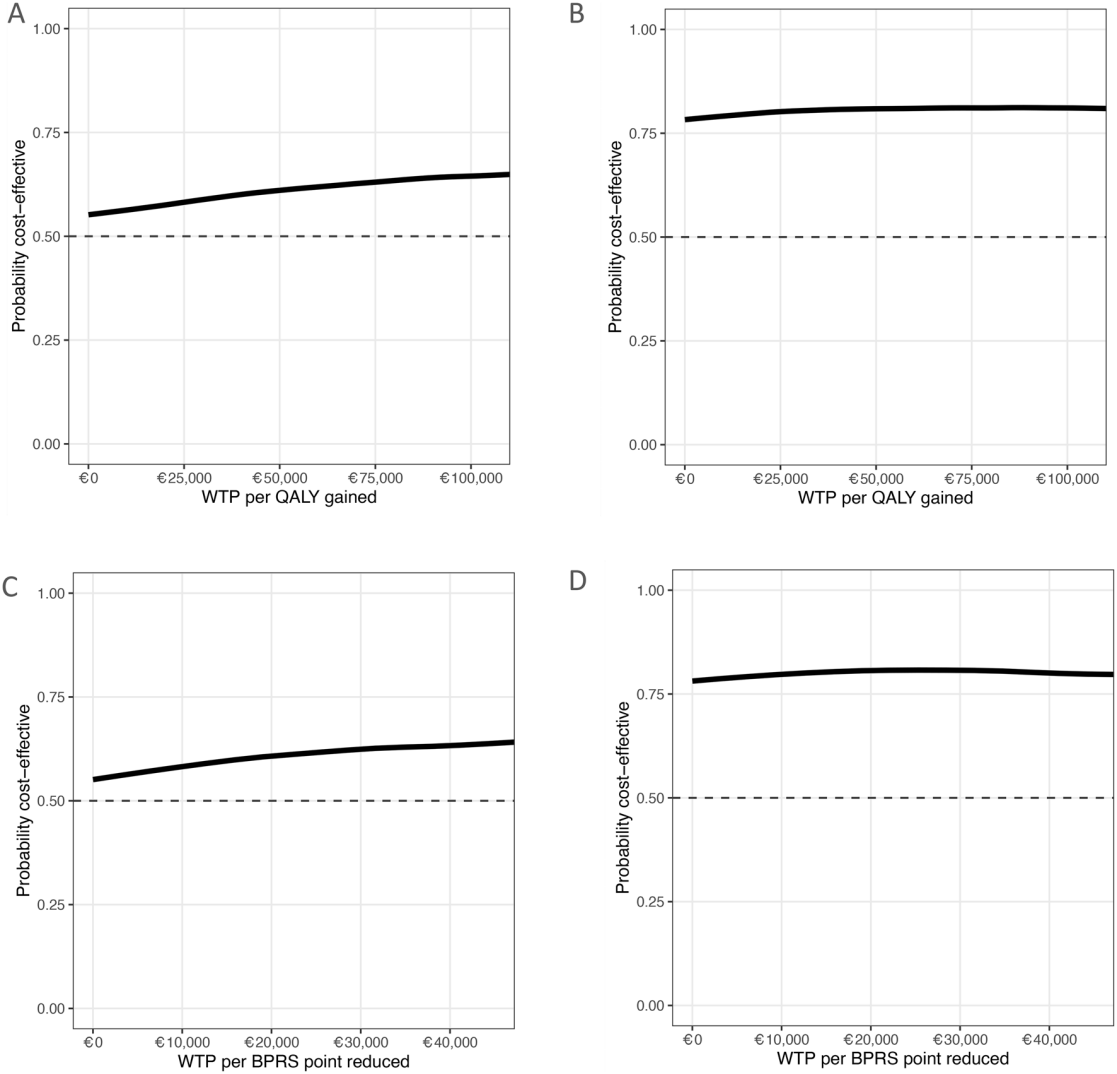
Cost-effectiveness acceptability curves from different cost perspectives and effects. Four cost-effectiveness acceptability show the probability that IHT is more cost-effective than CAU as a function of the willingness to pay (WTP) for one additional quality adjusted life years (QALY) or one additional change in brief psychiatric rating scale (BPRS) score. The indifference point is set at a probability of 0.5 on the vertical axis. Above this indifference point, IHT has a better likelihood to be preferred over CAU regarding cost-effectiveness

In Fig. 3, CEACs were drawn. These curves show the probability of IHT being more cost-effective than CAU as a function of the WTP for one additional QALY or one point improvement in BPRS score. From the societal perspective (Fig. 3A and C), and under the conservative assumption that there is no additional WTP for extra QALYs or a BPRS score reduction compared to the current situation under CAU, the probability that IHT will be cost-effective is > 50%. From the health care perspective (Fig. 3B and D), this probability is > 75% under the same conservative WTP threshold. The results from both perspectives were relatively stable under varying WTP levels, with only slightly higher cost-effectiveness probabilities when assuming higher WTP thresholds for QALYs and no observable change in probabilities for BPRS score reduction.

### Sensitivity analysis

The results of the sensitivity analysis, performed to assess the sensitivity of our findings to misspecification of costs for both QALYs and change in BPRS score, are presented in a tornado plot in Supplementary Material Fig. 1. The impact on the ICERs of a hypothetical − 20 to + 20% misspecification in societal or health care costs was limited. However, productivity costs were found to be impacted the most by any potential misspecification based on these analyses. If productivity costs were 20% lower than were assumed in our base case model, the ICER would have been lower and IHT would have been even more attractive in terms of the represented effects and costs.

## Discussion

This study evaluated the cost-utility and cost-effectiveness of IHT in comparison to CAU for patients experiencing an acute mental health crisis. From a societal perspective, IHT appears to have lower costs on average, although differences in costs are small and not statistically significant (mean difference of €-557; 95% CI − 9923 to 8237). Also, the effect differences between the two conditions are small (QALYs: mean difference: 0.02; 95% CI − 0.05 to 0.09; BPRS: mean difference: 0.04; 95% CI − 0.14 to 0.22) and not statistically significant.

The higher the WTP for a QALY, the more attractive IHT is compared to CAU. Under the NICE guidelines, which stipulate a maximum WTP of £30,000 (€35,000) per QALY [39], IHT could potentially be considered more cost-effective compared to CAU with a likelihood of only 55–60%, based on the cost-effectiveness acceptability curves. Yet, according to the Netherlands National Health Care Institute, for interventions addressing disorders with a high-disease severity, such as acute potentially life-threatening mental health crises, the WTP is €50,000 to €80,000 per QALY [40]. At this WTP level, the probability that IHT is better value for money than CAU increases somewhat with a cost-effectiveness likelihood of 60–65%.

From a health care perspective, the results lend stronger support to the cost-effectiveness of IHT over CAU. Here, the cost-utility analysis resulted in a negative ICER of €-22,759, which indicates that IHT probably leads to lower health care costs whilst achieving better health outcomes [39]. Here, the likelihood that IHT is more attractive than CAU from a cost-effectiveness perspective is > 75%, even with no WTP for additional QALYs. The directions of the results from the analyses with more proximal mental health outcomes (BPRS) were similar. The results of the sensitivity analysis indicate that our results were more sensitive to misspecification of productivity costs than to a misspecification of health care costs.

### Comparison to other studies

Our findings regarding difference in costs from a health care perspective seem to be similar to the study conducted by McCrone et al. (12) and Kilian et al. [41]. Using data from a controlled trial, McCrone et al. reported that costs for the IHT group were significantly lower compared to standard services across the six-month follow-up period (difference: £2,438; 90% CI, £937 to £3,922). A prospective cohort study performed in the rural areas of Germany [41] using reimbursement cost data found that IHT was significantly less expensive than CAU (€7,151 less costly per treatment episode). Moreover, Kilian and colleagues revealed a significant net monetary benefit for one unit change of depressive symptoms at a maximum WTP of €0 and €100. In addition, they found a net monetary benefit for one unit change in the general level of clinical and functional impairment (measured with the HoNOS) at a maximum WTP of €1,000.

### Relative difference in costs between societal and health care perspective

The key reason why the societal costs are higher in the IHT group is the relatively higher productivity costs. This finding was not something we anticipated. To help interpret this finding, we evaluated why participants in IHT could have had relatively higher productivity costs over the 52-week follow-up period. We found that participants in the IHT group had somewhat higher employment rates and were in treatment for more weeks in total than CAU participants, and therefore potentially higher productivity losses were accrued. Although our data support these hypotheses to some extent, more research is needed to clarify the mechanism behind the higher productivity losses amongst IHT patients.

### Limitations and strengths

The findings of this study should be interpreted in the light of its limitations and strengths. First, IHT was completely integrated into the mental health services, allowing for the provision of personalised care. As a result of this integration, it was not possible to separate the costs of IHT treatment from the overall costs of other mental health services. In the future research, the cost of an intervention that is fully integrated into health care services should be considered beforehand. Second, we used reference costs from the Netherlands. Although all costs were converted to OECD standard purchasing power parities for the index year 2019, the applicability of the costs presented in this economic evaluation may vary across countries. Third, a potential limitation to the generalisability of this study’s findings is that patients with a primary addiction problem and severe mental illness patients receiving ongoing outpatient care ((F)ACT) have not been included in our study; as in other areas in the Netherlands and in other countries, patients receiving FACT care could receive IHT care. Fourth, although the analysis took the societal perspective, we did not apply a monetisation of the psychological burden of informal caregivers in the presented analyses. Fifth, the study was not primarily powered to perform an economic evaluation –as there was too limited data available to formulate precise hypotheses regarding to be expected cost and effect differences, and as due to skewed cost data, large samples are generally needed for hypothesis testing in economic evaluations. Sixth, in some situations, the 95% confidence intervals of the ICER could not be determined (indicated by positive infinite values), as a result of a positive cost difference (the numerator of the ICER) and a negative effect difference (the denominator of the ICER). When higher costs are associated with lower effects, an ICER is not meaningful. Finally, although the study had a relatively long follow-up period of 52 weeks, it is possible that longer-term effects could have occurred amongst the studied population; those effects were not covered or modelled in this economic evaluation.

The strength of the study is its randomised design—many of the previous studies on IHT employed a naturalistic cohort design. Another strength is the extensive assessment of baseline and follow-up health care effects and costs in a vulnerable patient population, with high measurement compliance rates.

### Implications for clinical practice and future research

IHT has a fair probability of being more cost-effective than CAU, although differences are small from a societal perspective. However, the question remains whether IHT should be widely implemented despite the fact—based on our data—that no major economic advantages and no differences in QALYs or symptomatic change were found from a societal perspective. Nevertheless, this study shows that IHT enables professionals working in crisis care to efficiently deploy their resources by initially offering crisis treatment, with effects similar to those of usual crisis care interventions and probably at lower costs. Moreover, the results for the health care perspective also favour IHT over CAU, and here the effects are more pronounced. In our clinical experience, many patients have a preference for treatment at home rather than hospitalisation. IHT offers a care modality that many patients prefer with no economic disadvantages.

Future research should focus on how the cost-effectiveness of IHT can be further improved. Potential avenues to do so would include being able to predict which patients are most likely to benefit from IHT before they are assigned to IHT and supporting IHT patients to return to work more effectively to address the higher productivity costs in IHT. The second focus for future research should be identifying the impacts of IHT across different individuals, as in this study additional subgroup analyses were not performed due to the diversity of the included patients and thus smaller subgroups. Moreover, we recommend that future research consider possible distributional effects and describe them in their health economic analysis plan.

## Supplementary Information

Below is the link to the electronic supplementary material.

Supplementary file1 (DOCX 23 KB)

Supplementary file2 (DOCX 44 KB)

## Data Availability

The data that support the findings of this study are available from MB, upon reasonable request. The data are not publicly available due to their containing information that could compromise the privacy of research participants.
