# Supplementary material for: Economic evaluation of intensive home treatment in comparison to care as usual alongside a randomised controlled trial
Source: Eur J Health Econ. 2024 Apr 10;26(1):23–34. doi: 10.1007/s10198-024-01675-1 (PMC11743399; doi:10.1007/s10198-024-01675-1)
Supplement: Supplementary file 1 — Supplementary file1 (DOCX 23 KB) [file 10198_2024_1675_MOESM1_ESM.docx]

|  |  | IHT (n = 146) | | CAU (n = 52) | |
| --- | --- | --- | --- | --- | --- |
| Measurement | Time (weeks) | Mean | s.d. | Mean | s.d. |
| Effects | | | | | |
| BPRS total score | Baseline | 1.83 | 0.42 | 1.75 | 0.41 |
|  | 6 | 1.56 | 0.43 | 1.57 | 0.44 |
|  | 26 | 1.58 | 0.44 | 1.61 | 0.50 |
|  | 52 | 1.66 | 0.48 | 1.62 | 0.47 |
| QALYs | Baseline | 0.00 | 0.00 | 0.00 | 0.00 |
|  | 6 | 0.14 | 0.04 | 0.13 | 0.04 |
|  | 26 | 0.40 | 0.10 | 0.38 | 0.11 |
|  | 52 | 0.78 | 0.19 | 0.77 | 0.22 |
| Cumulative costs in € | | | | | |
| Total societal costs | Baseline | 0.00 | 0.00 | 0.00 | 0.00 |
|  | 6 |  |  |  |  |
|  | 26 | 27909.83 | 18216.03 | 27964.07 | 16500.61 |
|  | 52 | 44012.54 | 28801.94 | 44628.74 | 28869.93 |
| Total health care costs | Baseline | 0.00 | 0.00 | 0.00 | 0.00 |
|  | 6 | 9058.48 | 4359.10 | 9740.65 | 4571.63 |
|  | 26 | 23290.53 | 17807.36 | 24750.25 | 16157.43 |
|  | 52 | 34997.68 | 27197.60 | 38444.93 | 28526.71 |
| **Supplementary Table 1. Intervention effects and costs per outcome assessment wave**  All results are based on multiple imputed data. IHT = intensive home treatment; CAU = care as usual; BPRS = Brief Psychiatric Rating Scale; Quality adjusted life years (QALYs) averaged cumulative number of QALYs since baseline per patient at the end of each time point, with QALYs based on the Dutch European Quality of Life-5 Dimensions score; At 6 weeks the Trimbos Questionnaire for Costs associated with Psychiatric illness was not administered, consequently, societal costs could not be calculated. | | | | | |
