# Supplementary material for: Economic evaluation of intensive home treatment in comparison to care as usual alongside a randomised controlled trial
Source: Eur J Health Econ. 2024 Apr 10;26(1):23–34. doi: 10.1007/s10198-024-01675-1 (PMC11743399; doi:10.1007/s10198-024-01675-1)
Supplement: Supplementary file 2 — Supplementary file2 (DOCX 44 KB) [file 10198_2024_1675_MOESM2_ESM.docx]

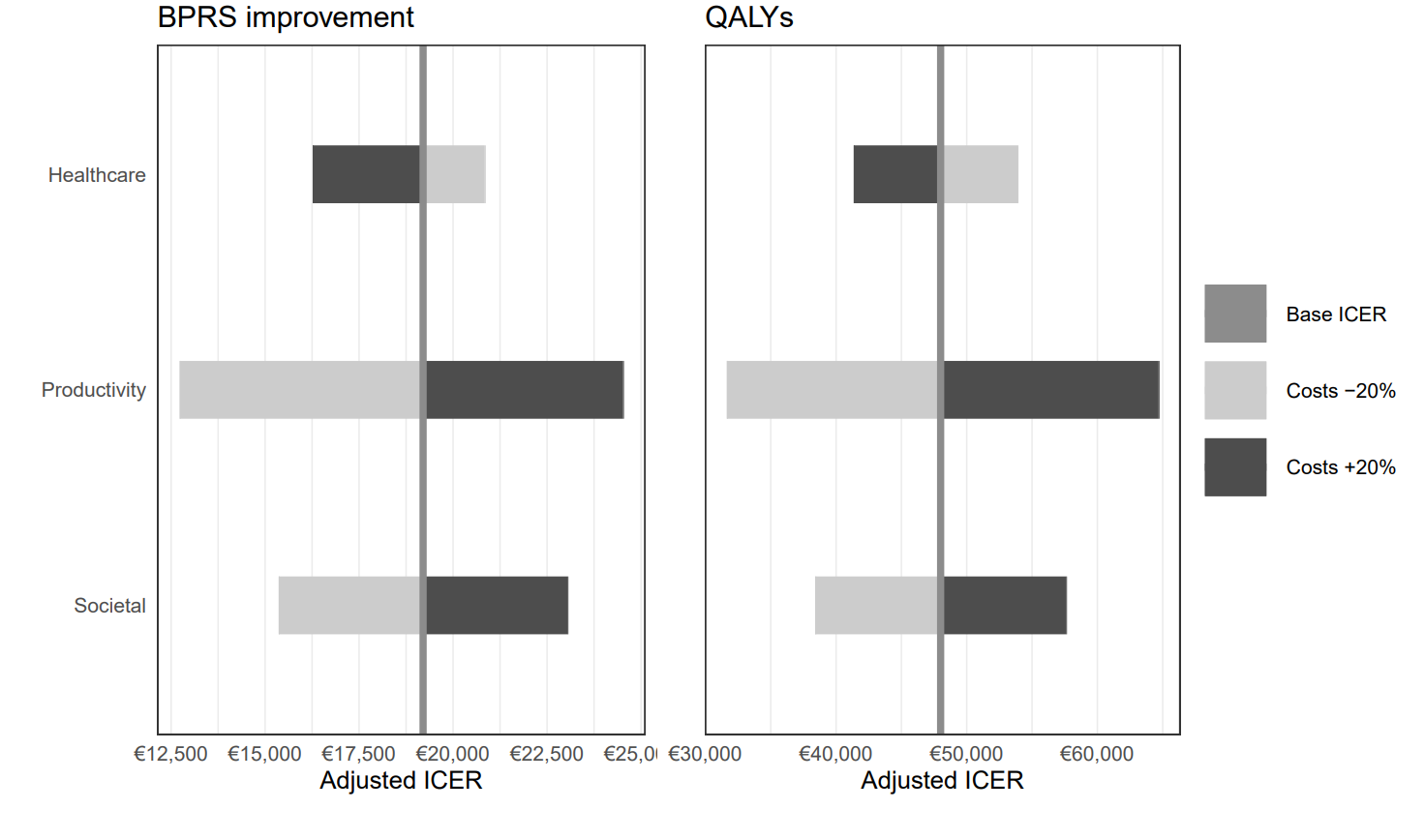
 **Supplementary Fig. 1.** **Results of the sensitivity analysis**

This tornado plot indicates what the ICER with brief psychiatric rating scale (BPRS) score improvement or quality adjusted life years (QALY) gained as an outcome would have been if the cost drivers on the vertical axis had been 20% higher or 20% lower than in the base case scenario. The ICER in the base case scenario is indicated by the vertical grey line that has a cross-section with the horizontal axis near the value of € 19203 for the BPRS and € 48003 for the QALYs. The cost model is most sensitive to a misspecification productivity costs (vertical axis).
